# Multi-system aging vulnerability associated with prolonged violence exposure: evidence from a Colombian sample

**DOI:** 10.3389/fnhum.2026.1840131

**Published:** 2026-08-21

**Authors:** Eduar Herrera, Alvaro Barrera-Ocampo, Willian Orlando Castillo Ordoñez

**Affiliations:** 1 Departamento de Estudios Psicológicos, Universidad Icesi, Cali, Colombia; 2 Grupo Natura, Centro BioInc, Departamento de Ciencias Farmacéuticas, Biomédicas y Veterinarias, Facultad Barberi de Ingeniería, Diseño y Ciencias Aplicadas, Universidad Icesi, Cali, Colombia; 3 Facultad de Ciencias Naturales, Exactas y de la Educación, Departamento de Biología, Universidad del Cauca, Popayán, Colombia

**Keywords:** accelerated aging, brain health, cognitive, cortisol, neuroendocrine, social factors, violence exposure

## Abstract

Exposure to violence is a major social determinant that can influence aging trajectories and brain health. In Colombia, a country affected by decades of armed conflict, comprehensive assessment models are needed to capture the multiple factors that shape health and may accelerate aging. This study examined whether long-term exposure to violence is associated with greater multisystem aging vulnerability across cognitive, mental health, social, and neuroendocrine domains in a Colombian cohort. Fifty participants were included: 31 victims of armed conflict (VAC-COL) and 19 control participants, matched for age and education. Assessments included global cognition (MoCA), executive functioning (IFS), symptoms of anxiety, depression, and post-traumatic stress disorder, daily functioning (FAST), perceived stress (PSS), everyday discrimination related to violence (EDVC), and plasma cortisol levels. A Multi-System Aging Vulnerability Index (MAVI) was constructed by standardizing and integrating measures across these domains. The VAC-COL group showed significantly higher MAVI scores, greater mental health symptom burden, higher cortisol levels, greater perceived stress and discrimination, and poorer daily functioning. In addition, a longer duration of violence exposure was positively associated with higher MAVI scores. Although preliminary, these findings suggest that prolonged exposure to violence in Colombia is associated with multisystem deterioration and increased aging-related vulnerability. The results underscore the importance of assessment frameworks that extend beyond isolated clinical symptoms and recognize violence as a critical determinant of brain health and aging-related vulnerability.

## 1 Introduction

Accelerated aging refers to a condition in which biological and functional decline occur at a rate that exceeds what would be expected based on chronological age (Salazar-Londoño et al., 2025; Hooten et al., 2022; Hamczyk et al., 2020). Beyond molecular and cellular markers, accelerated aging can also be conceptualized as a multidimensional process involving cognitive deterioration, emotional dysregulation, neuroendocrine imbalance, and psychosocial vulnerability (Zannas, 2024). The relationship between environmental exposures and biological functioning can be understood through the concept of the exposome, which encompasses the totality of environmental influences encountered across the lifespan that affect brain health (Vermeulen et al., 2020). Environmental toxins and adverse social conditions can amplify stress-related processes mediated by socioeconomic factors (Vermeulen et al., 2020; Landrigan et al., 2023). Through cumulative biological embedding mechanisms, including epigenetic modulation and stress-related neuroendocrine alterations, the exposome may shape brain health over time, thereby increasing vulnerability to subsequent stressors (Ibanez et al., 2024a,b; Robinson et al., 2026).

Population aging is a global phenomenon (Economic UNDo Affairs S, 2023). As life expectancy increases, so does the prevalence of cognitive decline associated with genetic, epigenetic, physical, and social determinants of health (Galluzzi et al., 2018; Coronel-Oliveros et al., 2025). Stress-related factors and mental health conditions contribute substantially to cognitive vulnerability (Gulpers et al., 2019; Günak et al., 2020; Lei et al., 2025; Scott et al., 2015). Prolonged exposure to violence is a particularly potent chronic stressor that can disrupt neurobiological systems and cognitive processes, thereby increasing the risk of mild cognitive impairment (MCI) and neurodegenerative disorders, including Alzheimer's disease (Scott et al., 2015; Justice, 2018; Gupta and Chaudhuri, 2025; Sahin and Aslan, 2024; Veggi and Roveta, 2025; Wadhwa et al., 2018). Such exposure has been linked to alterations in brain structure and function, cognitive decline, and heightened susceptibility to age-related conditions. Neurobiological models suggest that violence-related chronic stress may lead to sustained activation of the hypothalamic-pituitary-adrenal (HPA) axis, resulting in elevated cortisol levels (Lei et al., 2025). Although this response is initially adaptive, prolonged activation can produce hormonal dysregulation and structural brain changes (Scott et al., 2015; Ouanes and Popp, 2019). These alterations have been associated (James et al., 2023) with impairments in attention (Leng et al., 2013), implicit and explicit memory (Olver et al., 2015), decision-making, emotional regulation, and fear processing (Mavrych et al., 2025; Lupien et al., 2009). Chronic violence exposure may particularly affect amygdala-prefrontal circuits, which play a central role in emotional regulation and executive control (Mavrych et al., 2025; Keding et al., 2025).

From this perspective, chronic exposure to violence may compromise multiple dimensions of brain health, extending beyond mental health symptoms to affect cognitive, emotional, physical, psychosocial, and biological functioning across the lifespan (Rost et al., 2023). Structural inequities, including socioeconomic inequality and racism, may further exacerbate stress and intensify the adverse effects of violence on brain health (Grasser and Jovanovic, 2022). Colombia is among the countries with the highest levels of income inequality worldwide, as reflected by the Gini coefficient (Government of Colombia Ministry of Health Social Protection, 2019; Bank, 2019), and has endured a protracted armed conflict lasting more than six decades (Guerrero and Fandiño-Losada, 2017; Marroquín Rivera et al., 2020; Arranz et al., 2025). Sustained exposure to such conditions provides a critical context for examining the cumulative impact of violence on brain health and aging (Cardona et al., 2025a,b).

Research on populations exposed to chronic violence has highlighted the potential role of structural violence in accelerated aging. However, most studies have relied on isolated clinical symptoms or single biological markers. While informative, these approaches may underestimate the cumulative burden of chronic stress across interacting cognitive, psychological, social, and physiological systems. This limitation constrains our understanding of the multidimensional nature of accelerated aging. Integrative approaches that simultaneously assess cognitive, mental health, social, and neuroendocrine domains remain scarce, particularly in populations experiencing prolonged violence exposure. Moreover, much of the existing research has been conducted in cultural and socioeconomic contexts distinct from Colombia. The country's persistent structural inequalities and sustained exposure to armed conflict provide an important setting in which to examine how chronic violence contributes to cognitive and biological changes across the lifespan, potentially accelerating aging processes.

To address this gap, this study adopts a dimensional framework that conceptualizes accelerated aging from a brain health perspective. We integrate measures of cognitive performance, mental symptoms, stress, social adversity, and cortisol levels into a unified multidimensional index. Within this multidimensional framework, the biological domain is represented by neuroendocrine function, assessed using cortisol as an indicator of hypothalamic-pituitary-adrenal (HPA) axis activity. This approach is informed by theoretical models of allostatic load and biological stress, which describe how chronic adversity influences executive functioning, global cognition, emotional regulation, and hypothalamic-pituitary-adrenal axis activity. We hypothesized that individuals exposed to prolonged violence would exhibit: (Salazar-Londoño et al., 2025) poorer performance in global cognition and executive functioning; (Hooten et al., 2022) higher levels of stress-related symptoms, including post-traumatic stress disorder (PTSD) and perceived stress; and (Hamczyk et al., 2020) elevated cortisol levels relative to non-exposed controls. Additionally, we hypothesized that the cumulative duration of violence exposure would be positively associated with a MAVI. By adopting this multidomain perspective, the present study aims to move beyond symptom-specific analyses and toward a systems-level understanding of how prolonged violence exposure may be associated to aging-related vulnerability in brain health.

## 2 Materials and methods

### 2.1 Participants

The study included 50 participants with a mean age of 53.56 years (SD = 8.04) and a mean educational attainment of 12.08 years (SD = 4.55). Participants were classified into two groups: 19 control participants (CG) and 31 victims of armed conflict in Colombia (VAC-COL). Violence exposure status was established based on official registration in the Colombian Victims Unit registry and corroborated through structured interviews that assessed the duration and type of armed conflict-related experiences, such as forced displacement, direct exposure to violence, and threats.

Participants were recruited through nonprofit organizations and community leaders involved in programs supported by the Victims Unit of the Department of Valle del Cauca, Colombia. All participants completed standardized cognitive assessments and self-report questionnaires. In addition, peripheral blood samples were collected for cortisol analysis. Exclusion criteria included a current diagnosis of major neurological disorders, such as stroke or epilepsy, severe psychiatric disorders requiring hospitalization, including psychosis or bipolar disorder, substance dependence, or current use of systemic corticosteroid medication. Participants presenting with subclinical symptoms of anxiety, depression, or PTSD were not excluded, as these variables were examined dimensionally in subsequent analyses.

The study protocol was approved by the Ethics Committee of Universidad Icesi, and all participants provided written informed consent in accordance with the Declaration of Helsinki. The sample size was determined based on feasibility considerations within the target population. Given the exploratory and multidimensional nature of the study, effect sizes were emphasized alongside statistical significance. The studies involving human participants were approved by the Human Research Ethics Committee at Universidad Icesi. The research was conducted in accordance with local legislation and institutional requirements. All participants provided written informed consent before participation.

### 2.2 Measures

#### 2.2.1 Cognitive and clinical measures

A battery of standardized instruments and validated questionnaires was used to assess cognitive functioning, psychological symptoms, and daily functioning. Global cognitive performance was evaluated using the Montreal Cognitive Assessment (MoCA), a brief and sensitive instrument for detecting MCI in diverse populations (Nasreddine et al., 2019). Executive functioning was assessed with the INECO Frontal Screening (IFS), a neuropsychological tool designed to detect executive deficits associated with frontal lobe dysfunction (Torralva et al., 2010). Symptoms of anxiety and depression were assessed using the Hopkins Symptom Checklist-25 (HSCL-25) (Blevins et al., 2015). Post-traumatic stress symptoms were assessed with the PTSD Checklist for DSM-5 (PCL-5), a 20-item self-report instrument widely used in trauma-exposed populations.

Functional impairment in daily life was evaluated using the Functioning Assessment Short Test (FAST) (Rosa et al., 2007), a clinician-administered instrument that assesses autonomy, occupational functioning, cognitive functioning, financial management, interpersonal relationships, and leisure activities. Psychosocial stressors were assessed using the Everyday Discrimination Related to Violence in Colombia (EDVC) scale (Williams et al., 1997) and the Perceived Stress Scale (PSS) (Cohen et al., 1994), a widely used and well-validated instrument for measuring perceived stress in epidemiological research.

#### 2.2.2 Cortisol measurement in plasma

Peripheral blood samples were collected between 8:00 and 10:00 a.m. by a trained healthcare professional, a nurse or a clinical laboratory professional specialized in bacteriology, to minimize the influence of diurnal variation in cortisol levels. All participants were instructed to fast overnight before sample collection. Blood was collected into heparin-treated tubes, immediately labeled, and transported on ice to the laboratory.

Samples were centrifuged at 2,000 x g for 15 min at room temperature (25 °C). Plasma was carefully extracted, aliquoted into 1.5 mL microtubes, and stored at −80 °C until further analysis. Cortisol concentrations were measured using a commercially available competitive enzyme-linked immunosorbent assay kit (Abcam, catalog no. ab108665, Cambridge, UK), according to the manufacturer's instructions. Briefly, 20 μL of plasma samples, standards, and controls were added to microplate wells precoated with anti-cortisol IgG, followed by the addition of 200 μL of cortisol-HRP conjugate. Plates were covered with foil and incubated for 1 h at 37 °C. Wells were washed three times with 1x wash buffer before the addition of 100 μL of TMB substrate. After a 15-min incubation at room temperature in the dark, the enzymatic reaction was stopped by adding 100 μL of stop solution. Absorbance was read at 450 nm using a microplate reader (Synergy H1, BioTek, Winooski, VT, USA) within 5 min of stopping the reaction. Cortisol concentrations were determined by interpolating from a four-parameter logistic standard curve generated using known concentrations ranging from 0–500 ng/mL. The assay's sensitivity was 2.44 ng/mL, and both intra- and inter-assay coefficients of variation were below 9.8%, indicating high analytical precision (Başoglu et al., 2005; Abcam, 2018).

## 3 Statistical analysis

Statistical analyses were conducted in four sequential stages: (Salazar-Londoño et al., 2025) between-group comparisons to identify baseline differences across all variables; (Hooten et al., 2022) multivariate modeling to determine whether cognitive differences persisted after adjusting for mental health; (Hamczyk et al., 2020) construction of a MAVI to capture cumulative effects across domains; and (Zannas, 2024) simple linear regression analysis to assess whether the duration of violence exposure predicts the MAVI. Descriptive statistics, including means and standard deviations, were calculated for all variables. Between-group comparisons were performed using independent-samples *t*-tests, with Welch's correction applied when variances were unequal. Normality assumptions were evaluated using Shapiro-Wilk tests and visual inspection of Q-Q plots. Welch's correction was applied when appropriate to account for unequal variances. Effect sizes were calculated using Cohen's *d* to quantify the magnitude of group differences.

A one-way multivariate analysis of covariance (MANCOVA) was conducted to assess the effect of violence exposure on two cognitive outcomes, global cognition (MoCA) and executive functioning (IFS), while controlling for symptoms of anxiety, depression, and PTSD. Homogeneity of variance-covariance matrices was assessed using Box's M test, and multicollinearity among covariates was evaluated using variance inflation factors. *Post hoc* power analysis was conducted for the primary outcome using a two-tailed independent-samples *t*-test. The analysis was based on the observed group sizes (control: *n* = 19, violence-exposed group: *n* = 31), the observed standardized mean difference (Cohen's *d* = 1.67), and an alpha level of 0.05. Statistical power (1- β) was estimated using the noncentral *t*-distribution to assess whether the available sample size was adequate for detecting the observed between-group difference. To control for multiple comparisons, the *p*-values for the seven between-group comparisons included in the MAVI were adjusted using the Benjamini-Hochberg false discovery rate (FDR) procedure. Statistical significance was assessed using the FDR-adjusted *p*-values (*q* < 0.05). All analyses were performed using R software (version 4.4.2).

### 3.1 Multi-system aging vulnerability index (MAVI**)**

To construct the MAVI, seven variables representing distinct domains were integrated: cortisol levels, global cognition (MoCA), executive functioning (IFS), PTSD symptoms, daily functioning (FAST), everyday discrimination (EDVC), and perceived stress (PSS). All variables were transformed into Z-scores using the pooled sample mean and standard deviation.

The MAVI was calculated using the following equation:


$$\begin{array}{l} \text{MAVI}\;=+\text{Z}\;\left(\text{Cortisol}\right)-\text{Z}\;\left(\;\text{IFS}\right)-\text{Z}\left(\text{MoCA}\right)+\text{Z}\left(\text{PTSD}\right)\\ \;+\text{Z}\left(\text{FAST}\right)+\text{Z}\left(\text{PSS}\right)+\text{Z}(\text{EDVC}) \end{array}$$

Higher MAVI scores indicate greater multidimensional deterioration associated with accelerated aging. The negative signs assigned to MoCA and IFS reflect the protective direction of these measures, as higher scores indicate better cognitive performance.

To our knowledge, no prior study has developed an identical composite index. However, this approach is supported by previous research employing similar indices and identifying the included domains as markers of accelerated aging and allostatic load (Seeman et al., 1997; Belsky et al., 2015; Karlamangla et al., 2014; Freni-Sterrantino et al., 2022; Han et al., 2019; Mikelsone et al., 2023).

#### 3.1.1 Construction of the **MAVI**

The MAVI was designed as a composite measure capturing cognitive, mental health, neuroendocrine, and social dimensions of aging-related decline. The included measures were selected based on prior evidence linking changes in these domains to accelerated aging and cumulative stress burden (Karlamangla et al., 2014; Han et al., 2019; Mikelsone et al., 2023). Equal weights were assigned to all components to minimize overfitting and to reflect the conceptual assumption that each domain contributes comparably to cumulative stress-related burden, consistent with traditional allostatic load indices (Seeman et al., 1997; Karlamangla et al., 2014).

The directionality of each indicator was defined a priori. Variables for which higher values indicated greater impairment were entered directly as *Z*-scores. For cognitive measures, in which higher scores indicate better performance, values were multiplied by −1 before inclusion. The final MAVI was obtained by summing all transformed *Z*-scores, resulting in a continuous index in which higher values indicate greater multidimensional decline and a more pronounced accelerated aging profile.

## 4 Results

### 4.1 Demographic, biological, mental health, social, and cognitive measures

Significant group differences were observed across clinical, social, physiological, and cognitive domains (Table 1). The groups did not differ significantly in age, *t*(48) = 1.38, *p* = 0.173, or years of education, *t*(48) = 1.55, *p* =0.127, indicating adequate demographic comparability between the violence-exposed and control groups.

**Table 1:** Demographic, biological, mental health, social, and cognitive measures.

	Control (*n* = 19)	VAC-COL (*n* = 31)	*d* Cohen	Control vs. VAC-COL	*p* ajustado FDR^*^
Demographics					
Age	55.4(6.52)	52.4(8.74)	0.40	*t*(48) = 1.38 *p* = 0.173	
Education	13.2(3.15)	11.4(5.16)	0.40	*t*(48) = 1.55; *p* = 0.127	
Sex (F/M)	7/12	9/22			
Time exposed o violence	n/a	17.42(6.07)			
Biological, mental health, social, and cognitive measures					
Cortisol (ng/mL)	6.50 (1.25)	7.4 (0,76)	1.00	*t*(48) = −3.05; *p* = 0.005	0.005241
Hopkins symptom checklist (HSCL-25)—anxiety	5.05 (2.39)	13.5 (7.82)	−1.30	*t*(48) = −5.44; *p* = 0.001	
Hopkins symptom checklist-25 (HSCL-25)—depression	5.42 (2.59)	15.7 (11.19)	−1.14	*t*(48) = −4.91; *p* = 0.001	
PTSD checklist (PCL)	4.58 (4.00)	21.4 (12.32)	−1.68	*t*(48) = −7.03; *p* = 0.001	0.000000132
Perceived stress scale (PSS)	20.89 (4.20)	28.00 (5.62)	−1.38	*t*(48) = −5.09; *p* = 0.001	0.0000152
Everyday discrimination (EDVC)	9.47(5.43)	15.06(6.87)	0.88	*t*(48) = −3.19; *p* = 0.004	0.003035
Functioning assessment short test (FAST)	7.21 (4.86)	18.97 (12.8)	−1.11	*t*(48) = −4.60; *p* = 0.001	0.0000548
MoCA total score	25.53(3.20)	20.03 (4.67)	1.32	*t*(48) = 4.93; *p* = 0.001	0.0000185
IFS total score	24.00 (2.21)	20.32 (1.92)	1.81	*t*(48) = 5.99; *p* = 0.001	0.00000303
Multi-system aging vulnerability index (MAVI)	1.53(0.93)	3.13 (0.99)	1.67	*t*(48) = −5.86; *p* = 0.001	

^*^Only for the variables included in the MAVI.

The violence-exposed group had significantly higher plasma cortisol levels and more severe symptoms of anxiety, *t*(48) = −3.05, *p* = 0.005, as well as higher levels of anxiety symptoms, *t*(48) = −5.44, *p* < 0.001; depressive symptoms, *t*(48) =-4.91, *p* < 0.001, and PTSD symptoms, *t*(48) =-7.03, *p* < 0.001. Significant group differences were also observed for perceived stress (PSS), *t*(48) =-5.09, *p* < 0.001; functional impairment (FAST), *t*(48) =-4.60, *p* < 0.001; and EDVC, *t*(48) =-3.19, *p* = 0.004. With respect to cognitive performance, the violence-exposed group demonstrated significantly lower scores in global cognition, as measured by the MoCA, *t*(48) = 4.93, *p* < 0.001, and executive functioning, as measured by the INECO IFS, *t*(48) = 5.99, *p* < 0.001. All between-group differences in the variables included in the MAVI remained statistically significant after applying the Benjamini-Hochberg false discovery rate correction (all FDR-adjusted *p*-values < 0.01).

### 4.2 Adjusted multivariate analysis

A one-way multivariate analysis of covariance (MANCOVA) was conducted with group (violence-exposed vs. control) as the independent variable and global cognition (MoCA total score) and executive functions (IFS) as dependent variables. Symptoms of anxiety, depression, and PTSD, as well as sex, were included as covariates. The overall MANCOVA was statistically significant, Wilks' λ = 0.615, *F*(2, 43) = 13.45, *p* < 0.001. Follow-up univariate ANCOVAs revealed significantly lower adjusted scores in the violence-exposed group for both global cognition [*F*(1, 44) = 14.80, *p* = 0.001, ηp^2^ = 0.25] and IFS [*F*(1, 44) = 22.41, *p* < 0.001, ηp^2^ = 0.34], indicating medium-to-large effect sizes.

Anxiety symptoms were not significantly associated with either MoCA, *F*(1, 44) = 0.15, *p* =0.704, ηp^2^ = 0.003, or IFS performance, *F*(1, 44) = 0.26, *p* = 0.615, ηp^2^ = 0.006. Similarly, depressive symptoms were not related to MoCA *F*(1, 44) = 0.002, *p* = 0.960, ηp^2^ = 0.001, or IFS scores, *F*(1, 44) = 0.23, *p* = 0.636, ηp^2^ = 0.005. PTSD symptoms were also not significantly associated with cognitive performance on either measure (MoCA: *F*(1, 44) = 1.09, *p* = 0.301, ηp^2^ = 0.024; IFS: *F*(1, 44) = 1.59, *p* = 0.214, ηp^2^ = 0.035). Likewise, sex was not significantly associated with either MoCA performance, *F*(1, 44) = 1.40, *p* = 0.244, ηp^2^ = 0.031, or IFS performance, *F*(1, 44) = 1.38, *p* = 0.247, ηp^2^ = 0.030.

Overall, these findings indicate that group differences in cognitive performance remained significant after controlling for current anxiety, depression, and PTSD symptoms, and sex, suggesting that observed cognitive alterations could not be fully explained by current symptom severity or differences in sex distribution.

### 4.3 Multi-system aging vulnerability index (MAVI)

Based on the variables presented in Table 1, a MAVI was calculated by standardizing and integrating indicators across four domains: cognitive (MoCA, IFS), mental health (PTSD symptoms, FAST), physiological (plasma cortisol), and social (everyday discrimination and perceived stress). The violence-exposed group had significantly higher MAVI scores than the control group, *t*(48) =-5.86, *p* < 0.0001, Cohen's *d* = 1.67, indicating a large effect size. Higher MAVI values reflect greater multidimensional deterioration across cognitive, psychological, social, and physiological domains, consistent with greater aging-related vulnerability. A *post hoc* power analysis, considering the observed between-group difference in the MAVI (Cohen's *d* = 1.67), the sample sizes (*n* = 19 and *n* = 31), and a two-tailed independent-samples *t*-test ( α = 0.05), indicated a statistical power of 99.99%.

### 4.4 Duration of violence exposure as a predictor of multi-system aging vulnerability

A multiple linear regression analysis was conducted to examine the association between duration of violence exposure and MAVI scores within the violence-exposed group, adjusting for sex. Model assumptions, including normality, homoscedasticity, and the absence of influential outliers, were assessed through residual diagnostics and were satisfactorily met. The regression analysis revealed a significant positive association between duration of violence exposure and accelerated aging, *F*(2, 47) = 26.92, *p* < 0.001. The model explained 53.4% of the variance in MAVI scores, *R*^2^ =0.534. The regression coefficient indicated that each additional year of exposure to violence was associated with a 0.35-unit increase in the MAVI (*B* = 0.35, 95% CI [0.25, 0.45], *p* < 0.001), after adjusting for sex. Sex was not significantly associated with MAVI scores (*B* = −1.96, 95% CI [−4.00, 0.07], *p* = 0.059). The regression intercept was also statistically significant (β =-3.73, *p* < 0.001), representing the estimated MAVI score at zero years of exposure. Taken together, these results indicate that prolonged exposure to armed conflict is associated with multidimensional differences across cognitive, psychological, social, and physiological domains, and that longer exposure duration is associated with greater cumulative stress-related burden. This association remained significant after adjustment for sex.

## 5 Discussion

To our knowledge, this is the first study conducted in Colombia to operationalize aging-related vulnerability through a multidimensional index integrating cognitive, mental health, social, functional, and neuroendocrine indicators in a population exposed to armed conflict. The observed pattern of findings indicates that prolonged violence exposure is associated with disturbances across several interrelated domains rather than with isolated clinical manifestations. Collectively, these results support a systems-level framework for examining the relationship between chronic exposure to violence and aging-related vulnerability in brain health.

### 5.1 Mental health, neuroendocrine, social, and cognitive alterations in individuals exposed to violence

Individuals exposed to armed conflict exhibited a broad pattern of multidomain alterations, including more severe symptoms of depression, anxiety, and PTSD; elevated cortisol concentrations; greater perceived stress and discrimination; increased functional impairment; and poorer cognitive performance. This constellation of findings is consistent with previous evidence indicating that chronic violence exposure is associated with disturbances across multiple interrelated psychological, cognitive, social, and physiological system (Tsunga et al., 2025). The observed neuroendocrine alterations are also consistent with research demonstrating that chronic stress can disrupt HPA axis function (Joseph and Whirledge, 2017), as proposed within allostatic load models (Ibanez et al., 2024a) and documented in trauma-exposed populations (Maciaszek et al., 2025).

Previous studies conducted in Colombia have described comparable clinical and cognitive patterns, including heightened anxiety, somatic symptoms (Londoño et al., 2012), and impairments in executive functioning (Villanueva-Bonilla et al., 2024; Valencia et al., 2025). Of particular relevance, the between-group differences in global cognition and executive functioning remained statistically significant after adjustment for symptoms of anxiety, depression, and PTSD. This persistence indicates that the cognitive differences observed in the violence-exposed group cannot be attributed solely to current emotional symptom severity and may reflect broader cumulative processes associated with prolonged stress exposure.

This interpretation is consistent with evidence suggesting that cognitive and neurophysiological alterations in chronically stressed populations may emerge, at least partly, through mechanisms that are not fully accounted for by concurrent emotional symptoms (Herrera et al., 2025). Models of stress-related neural remodeling propose that sustained HPA axis activation and inflammatory signaling may affect prefrontal and hippocampal circuits, thereby influencing executive and memory-related processes. Importantly, such neurobiological alterations may not be fully indexed by subjective symptom reports. Accordingly, the present findings support the direct assessment of cognitive functioning in populations exposed to violence rather than the assumption that cognitive outcomes represent secondary manifestations of anxiety, depression, or PTSD.

This issue is particularly relevant because exposure to violence frequently occurs in conjunction with social, structural, and environmental adversities. These co-occurring conditions may exert cumulative effects on biological systems through allostatic load mechanisms and may contribute to persistent physiological and cognitive alterations (Cardona et al., 2025a,b). From this perspective, clinical symptom measures alone may provide an incomplete representation of the cumulative burden associated with violence exposure. Comprehensive assessment models should therefore incorporate objective cognitive and physiological indicators in addition to conventional measures of psychological distress.

### 5.2 A multidimensional approach to multi-system aging vulnerability

The significantly higher MAVI scores observed in the violence-exposed group indicate a greater cumulative burden across cognitive, psychological, functional, social, and physiological domains. The index should not, however, be interpreted as a direct estimate of biological age. Rather, it constitutes an operational measure of multidomain vulnerability associated with prolonged stress exposure. This distinction is important because the MAVI is intended to characterize the convergence of alterations across interacting systems rather than to quantify the discrepancy between chronological and biological age.

The multidimensional approach adopted in this study is consistent with previous investigations that have used composite indicators to characterize cumulative vulnerability and multisystem dysregulation (Simons et al., 2021). It also extends symptom-specific approaches by incorporating exposure-related, cognitive, functional, social, and neuroendocrine measures into a single analytical framework. In this regard, the MAVI may provide a useful exploratory tool for examining how chronic adversity is associated with broader patterns of aging-related vulnerability (Robinson et al., 2026).

Equal weights were assigned to the individual components of the index. This parsimonious strategy is consistent with established approaches in allostatic load research, in which composite indices are frequently constructed without assigning differential weights a priori (Seeman et al., 1997; Karlamangla et al., 2014). Nevertheless, equal weighting assumes that each component contributes comparably to the underlying construct. Future studies should therefore compare alternative weighting procedures and formally evaluate the reliability, construct validity, internal structure, and predictive validity of the MAVI in larger and independent samples.

### 5.3 Duration of violence exposure and aging-related vulnerability

A longer duration of violence exposure was positively associated with higher MAVI scores, suggesting that cumulative exposure may be relevant to the magnitude of multidomain vulnerability. Prolonged violence exposure may be associated with sustained activation or dysregulation of stress-regulatory systems, particularly the HPA axis (Gupta and Morley, 2014; Jovanovic et al., 2017; Lemus-Roldan et al., 2025). Persistent alterations in these systems have been linked to allostatic load processes, including cortisol dysregulation and inflammatory changes, which may adversely affect brain structure, cognitive functioning, and emotional regulation (Teferi et al., 2026; Volarić et al., 2024).

The association with exposure duration further indicates that the temporal extent of adversity should be considered when examining the long-term consequences of armed conflict. Duration may represent an important dimension of cumulative stress burden that is not adequately captured by the mere classification of individuals as exposed or non-exposed. Accordingly, analyses restricted to dichotomous exposure categories may overlook meaningful variability in the intensity and persistence of violence-related experiences.

Although the regression model explained a substantial proportion of the variance in MAVI scores, this finding should be interpreted in the context of the exploratory design and limited sample size. The magnitude of the association may be sample-specific and requires confirmation in larger, more heterogeneous populations. Replication in longitudinal cohorts will be particularly important to determine whether exposure duration predicts subsequent changes in cognitive, neuroendocrine, functional, and psychological outcomes.

### 5.4 Limitations and future directions

Several limitations should be considered when interpreting the present findings. First, the cross-sectional design precludes causal inferences regarding the direction or temporal sequence of the observed associations between violence exposure and multidimensional aging-related vulnerability. Second, the relatively small sample size and imbalance in sex distribution limit the generalizability of the findings and may have contributed to an overestimation of the observed effect sizes. Although the post hoc power analysis indicated sufficient power to detect the primary between-group difference in MAVI scores, estimates derived from small samples may still be unstable and imprecise.

Consequently, these findings should be replicated in larger, more diverse, and independent cohorts to establish the robustness and generalizability of the observed associations. Third, reliance on a single neuroendocrine biomarker, cortisol, cannot capture the full complexity of multisystem biological aging processes, including inflammatory, metabolic, and epigenetic alterations. Fourth, although the composite index was theoretically grounded, its reliability, validity, and internal structure require evaluation in larger and independent samples. Finally, the use of self-report measures for several domains may have introduced reporting bias.

Future longitudinal studies incorporating multimodal biomarkers, neuroimaging measures, and established indicators of biological aging will be essential to determine whether the observed multidimensional profile predicts subsequent aging trajectories, cognitive decline, or neurodegenerative outcomes.

## 6 Conclusion

This study provides preliminary evidence that exposure to armed conflict in Colombia is associated with multidimensional differences across cognitive functioning, mental health, neuroendocrine regulation, social stress, and daily functioning. The positive association between the duration of violence exposure and the MAVI suggests that cumulative violence exposure is associated with greater aging-related vulnerability across interacting cognitive, psychological, social, functional, and physiological domains. Although the proposed index does not directly estimate biological age, it provides a multidomain framework for characterizing cumulative stress-related burden beyond symptom-specific approaches. These findings underscore the importance of incorporating cognitive and physiological indicators into comprehensive assessment models and identify prolonged violence exposure as a factor associated with long-term brain health vulnerability.

## Data Availability

The raw data supporting the conclusions of this article will be made available by the authors, without undue reservation.

## References

[B1] Abcam (2018). Cortisol ELISA Kit (ab108665): A Competitive Immunoenzymatic Assay for the Quantitative Measurement of Cortisol in Serum and Plasma Version 4. Cambridge: Abcam.

[B2] Arranz J. Ferrer R. Zhu N. Rubio-Guerra S. Rodríguez-Baz Í. Arriola-Infante J. E. . (2025). Prospective evaluation of plasma pTau217 stability for the detection of Alzheimer's disease in a tertiary memory clinic. Alzheimer's Res. Ther. 17:150. doi: 10.1186/s13195-025-01779-7 40618130 PMC12228402

[B3] Bank W. (2019). Poverty and Equity Brief Colombia. Washington, DC: World Bank.

[B4] Başoglu M. Livanou M. Crnobarić C. Frančišković T. Suljić E. Ð*urić D. . (2005). Psychiatric and cognitive effects of war in former Yugoslavia: association of lack of redress for trauma and posttraumatic stress reactions. Jama 294, 580–590. doi: 10.1001/jama.294.5.580 16077052

[B5] Belsky D. W. Caspi A. Houts R. Cohen H. J. Corcoran D. L. Danese A. . (2015). Quantification of biological aging in young adults. Proc. Natl. Acad. Sci. 112, E4104–E10. doi: 10.1073/pnas.1506264112 26150497 PMC4522793

[B6] Blevins C. A. Weathers F. W. Davis M. T. Witte T. K. Domino J. L. (2015). The posttraumatic stress disorder checklist for DSM-5 (PCL-5): development and initial psychometric evaluation. J. Trauma. Stress 28, 489–498. doi: 10.1002/jts.22059 26606250

[B7] Cardona J. F. Santamaría-García H. Ibáñez A. (2025a). The biological burden of conflict across populations worldwide. Clin. Transl. Med. 16:e70574. doi: 10.1002/ctm2.70574 41454480 PMC12743140

[B8] Cardona J. F. Trujillo-Llano C. Miller J. Ibañez A. (2025b). Address Colombia's brain-health crisis. Nature 643:336. doi: 10.1038/d41586-025-02155-z 40629117

[B9] Cohen S. Kamarck T. Mermelstein R. (1994). “Perceived Stress Scale,” in Measuring Stress: A Guide for Health and Social Scientists, Vol. 10, eds. S. Cohen, R. C. Kessler, and L. U. Gordon (New York, NY: Oxford University Press), 1–2.

[B10] Coronel-Oliveros C. Moguilner S. Hernandez H. Cruzat J. Baez S. Medel V. . (2025). Diversity-sensitive brain clocks linked to biophysical mechanisms in aging and dementia. Nat. Ment. Health. 3, 1214–1229. doi: 10.1038/s44220-025-00502-7 41929992 PMC13042324

[B11] Economic UNDo and Affairs S (2023). World Social Report 2023: Leaving No One Behind in an Ageing World. New York, NY: UN.

[B12] Freni-Sterrantino A. Prescott T. P. Ceely G. Glickman M. Holmes C. (2022). Assessments and developments in constructing a national health index for policy making, in the United Kingdom. arXiv preprint arXiv:221005154.

[B13] Galluzzi L. Yamazaki T. Kroemer G. (2018). Linking cellular stress responses to systemic homeostasis. Nat. Rev. Mol. Cell Biol. 19, 731–745. doi: 10.1038/s41580-018-0068-0 30305710

[B14] Government of Colombia and Ministry of Health and Social Protection (2019). Colombia Violence Against Children and Youth Survey. Bogotá: IOM.

[B15] Grasser L. R. Jovanovic T. (2022). Neural impacts of stigma, racism, and discrimination. Biol. Psychiatry 7, 1225–1234. doi: 10.1016/j.bpsc.2022.06.012 35811064

[B16] Guerrero R. Fandiño-Losada A. (2017). Is Colombia a violent country? Colomb. Med. 48, 9–11. doi: 10.25100/cm.v48i1.2979 PMC5438222 28559640

[B17] Gulpers B. J. Oude Voshaar R. C. van Boxtel M. P. Verhey F. R. Köhler S. (2019). Anxiety as a risk factor for cognitive decline: a 12-year follow-up cohort study. Am. J. Geriatr. Psychiatry. 27, 42–52. doi: 10.1016/j.jagp.2018.09.006 30409551

[B18] Günak M. M. Billings J. Carratu E. Marchant N. L. Favarato G. Orgeta V. . (2020). Post-traumatic stress disorder as a risk factor for dementia: systematic review and meta-analysis. Br. J. Psychiatry. 217, 600–608. doi: 10.1192/bjp.2020.150 32933591

[B19] Gupta D. Morley J. E. (2014). Hypothalamic-pituitary-adrenal (HPA) axis and aging. Compr. Physiol. 4, 1495–1510. doi: 10.1002/j.2040-4603.2014.tb00585.x 25428852

[B20] Gupta D. K. Chaudhuri A. (2025). The role of stress, inflammatory markers, and environmental determinants in cognitive decline and dementia: a systematic review of recent evidence. Muller J. Med. Sci. Res. 16, 53–60. doi: 10.4103/mjmsr.mjmsr_81_24

[B21] Hamczyk M. Nevado R. Barettino A. Fuster V. Andrés V. (2020). Biological versus chronological aging: JACC focus seminar. J. Am. Coll. Cardiol. 75, 919–930. doi: 10.1016/j.jacc.2019.11.062 32130928

[B22] Han L. K. Verhoeven J. E. Tyrka A. R. Penninx B. W. Wolkowitz O. M. Månsson K. N. . (2019). Accelerating research on biological aging and mental health: current challenges and future directions. Psychoneuroendocrinology 106, 293–311. doi: 10.1016/j.psyneuen.2019.04.004 31154264 PMC6589133

[B23] Herrera E. Gutierrez-Sterling D. Barrera-Ocampo A. Jaramillo J. O. Santamaría-García H. Birba A. . (2025). Impaired interoception in Colombian victims of armed conflict with PTSD: a preliminary HEP study. Front. Psychol. 16:1567574. doi: 10.3389/fpsyg.2025.1567574 40351583 PMC12061942

[B24] Hooten N. N. Pacheco N. L. Smith J. T. Evans M. K. (2022). The accelerated aging phenotype: the role of race and social determinants of health on aging. Ageing Res. Rev. 73:101536. doi: 10.1016/j.arr.2021.101536 34883202 PMC10862389

[B25] Ibanez A. Kringelbach M. L. Deco G. (2024a). A synergetic turn in cognitive neuroscience of brain diseases. Trends Cogn. Sci. 28, 319–338. doi: 10.1016/j.tics.2023.12.006 38246816 PMC11927795

[B26] Ibanez A. Melloni L. Swieboda P. Hynes W. Ikiz B. Ayadi R. . (2024b). Neuroecological links of the exposome and one health. Neuron. 112, 1905–1910. doi: 10.1016/j.neuron.2024.04.016 38723637 PMC11189719

[B27] James K. A. Stromin J. I. Steenkamp N. Combrinck M. I. (2023). Understanding the relationships between physiological and psychosocial stress, cortisol and cognition. Front. Endocrinol. 14:1085950. doi: 10.3389/fendo.2023.1085950 36950689 PMC10025564

[B28] Joseph D. N. Whirledge S. (2017). Stress and the HPA axis: balancing homeostasis and fertility. Int. J. Mol. Sci. 18:2224. doi: 10.3390/ijms18102224 29064426 PMC5666903

[B29] Jovanovic T. Vance L. A. Cross D. Knight A. K. Kilaru V. Michopoulos V. . (2017). Exposure to violence accelerates epigenetic aging in children. Sci. Rep. 7:8962. doi: 10.1038/s41598-017-09235-9 28827677 PMC5566406

[B30] Justice N. J. (2018). The relationship between stress and Alzheimer's disease. Neurobiol. Stress. 8, 127–133. doi: 10.1016/j.ynstr.2018.04.002 29888308 PMC5991350

[B31] Karlamangla A. S. Miller-Martinez D. Lachman M. E. Tun P. A. Koretz B. K. Seeman T. E. . (2014). Biological correlates of adult cognition: midlife in the United States (MIDUS). Neurobiol. Aging. 35, 387–394. doi: 10.1016/j.neurobiolaging.2013.07.028 24011541 PMC3830604

[B32] Keding T. J. Russell J. D. Zhu X. He Q. Li J. J. Herringa R. J. . (2025). Diverging effects of violence exposure and psychiatric symptoms on amygdala-prefrontal maturation during childhood and adolescence. Biol. Psychiatry 10, 450–462. doi: 10.1016/j.bpsc.2024.08.003 PMC11885587 39182725

[B33] Landrigan P. Symeonides C. Raps H. Dunlop S. (2023). The global plastics treaty: why is it needed? Lancet. 402, 2274–2276. doi: 10.1016/S0140-6736(23)02198-0 37863083

[B34] Lei A. A. Phang V. W. X. Lee Y. Z. Kow A. S. F. Tham C. L. Ho Y. C . (2025). Chronic stress-associated depressive disorders: the impact of HPA axis dysregulation and neuroinflammation on the hippocampus—a mini review. Int. J. Mol. Sci. 26:2940. doi: 10.3390/ijms26072940 40243556 PMC11988747

[B35] Lemus-Roldan K. Castorena Torres F. León Rojas D. Rodríguez-de-Ita J. (2025). From early adversity to neurodegeneration: stress biomarkers as predictive signals for lifespan brain health. Int. J. Mol. Sci. 26:11013. doi: 10.3390/ijms262211013 41303499 PMC12652871

[B36] Leng Y. Wainwright N. Hayat S. Stephan B. Matthews F. Luben R. . (2013). The association between social stress and global cognitive function in a population-based study: the European Prospective Investigation into Cancer (EPIC)-Norfolk study. Psychol. Med. 43, 655–666. doi: 10.1017/S0033291712001316 22687394

[B37] Londoño A. Romero P. Casas G. (2012). The association between armed conflict, violence and mental health: a cross sectional study comparing two populations in Cundinamarca department, Colombia. Confl Health. 6:12. doi: 10.1186/1752-1505-6-12 23244206 PMC3605190

[B38] Lupien S. J. McEwen B. S. Gunnar M. R. Heim C. (2009). Effects of stress throughout the lifespan on the brain, behaviour and cognition. Nat. Rev. Neurosci. 10, 434–445. doi: 10.1038/nrn2639 19401723

[B39] Maciaszek J. Alejnikowa J. Dybek A. Bloch M. Misiak B. (2025). Lower levels of positive affect and insomnia are associated with elevated allostatic load among Ukrainian refugees. Psychoneuroendocrinology 175:107387. doi: 10.1016/j.psyneuen.2025.107387 39985861

[B40] Marroquín Rivera A. Rincón Rodríguez C. J. Padilla-Muñoz A. Gómez-Restrepo C. (2020). Mental health in adolescents displaced by the armed conflict: findings from the Colombian national mental health survey. Child Adolesc. Psychiatry Ment. Health. 14:23. doi: 10.1186/s13034-020-00327-5 32467726 PMC7236943

[B41] Mavrych V. Riyas F. Bolgova O. Mohamed F. R. R. (2025). The role of basolateral amygdala and medial prefrontal cortex in fear: a systematic review. Cureus. 17:e78198. doi: 10.7759/cureus.78198 40026920 PMC11870299

[B42] Mikelsone M. Reine I. Tomsone S. Guð*mundsson H. Ivanovs A. Guð*mundsson H. S. . (2023). Construction of healthy aging index from two different datasets. Front. Public Health. 11:1231779. doi: 10.3389/fpubh.2023.1231779 37744491 PMC10513080

[B43] Nasreddine Z. S. Phillips N. A. Bédirian V. Charbonneau S. Whitehead V. Collin I. . (2019). The Montreal Cognitive Assessment, MoCA: A brief screening tool for mild cognitive impairment: Corrigendum. 10.1111/j.1532-5415.2005.53221.x 15817019

[B44] Olver J. S. Pinney M. Maruff P. Norman T. R. (2015). Impairments of spatial working memory and attention following acute psychosocial stress. Stress Health 31, 115–123. doi: 10.1002/smi.2533 24395182

[B45] Ouanes S. Popp J. (2019). High cortisol and the risk of dementia and Alzheimer's disease: a review of the literature. Front. Aging Neurosci. 11:435469. doi: 10.3389/fnagi.2019.00043 PMC6405479 30881301

[B46] Robinson H. Dave N. Barzilay R. Wagner A. Kells N. Keller A. S. . (2026). The effect of the “exposome” on developmental brain health and cognitive outcomes. Neuropsychopharmacology 51, 169–184. doi: 10.1038/s41386-025-02180-6 40764756 PMC12618635

[B47] Rosa A. R. Sánchez-Moreno J. Martínez-Aran A. Salamero M. Torrent C. Reinares M. . (2007). Validity and reliability of the functioning assessment short test (FAST) in bipolar disorder. Clin. Pract. Epidemiol. Mental Health. 3:5. doi: 10.1186/1745-0179-3-5 17555558 PMC1904447

[B48] Rost N. S. Salinas J. Jordan J. T. Banwell B. Correa D. J. Said R. R. . (2023). The brain health imperative in the 21st century—a call to action: the AAN brain health platform and position statement. Neurology. 101, 570–579. doi: 10.1212/WNL.0000000000207739 37730439 PMC10558159

[B49] Sahin S. K. Aslan E. (2024). Inflammation as a neurobiological mechanism of cognitive impairment in psychological stress. J. Integr. Neurosci. 23:101. doi: 10.31083/j.jin2305101 38812387

[B50] Salazar-Londoño S. Pérez-Foucrier V. Baldera J. P. Aarsland M. Venegas-Sanabria L. C. Borda M. G. . (2025). Temporal muscle thickness predicts change in nutritional markers in individuals at risk of dementia: insights from a 24-week longitudinal study. J. Aging Res. Lifestyle. 14:100023. doi: 10.1016/j.jarlif.2025.100023 PMC12357314 40821925

[B51] Scott S. B. Graham-Engeland J. E. Engeland C. G. Smyth J. M. Almeida D. M. Katz M. J. . (2015). The effects of stress on cognitive aging, physiology and emotion (ESCAPE) project. BMC Psychiatry. 15:146. doi: 10.1186/s12888-015-0497-7 26138700 PMC4490700

[B52] Seeman T. E. Singer B. H. Rowe J. W. Horwitz R. I. McEwen B. S. (1997). Price of adaptation—allostatic load and its health consequences: MacArthur studies of successful aging. Arch. Intern. Med. 157, 2259–2268. doi: 10.1001/archinte.1997.00440400111013 9343003

[B53] Simons R. L. Lei M. K. Klopack E. Beach S. R. . (2021). The effects of social adversity, discrimination, and health risk behaviors on the accelerated aging of African Americans: further support for the weathering hypothesis. Soc. Sci. Med. 282:113169. doi: 10.1016/j.socscimed.2020.113169 32690336 PMC7790841

[B54] Teferi H. M. Shelton R. C. Conneely K. N. De Vivo I. Factor-Litvak P. Kezios K. L. . (2026). Allostatic load and biological aging among middle aged adults. Psychoneuroendocrinology. 183:107661. doi: 10.1016/j.psyneuen.2025.107661 41223677

[B55] Torralva T. Roca M. Gleichgerrcht E. Lopez P. Manes F. (2010). INECO Frontal Screening (IFS): a brief, sensitive, and specific tool to assess executive functions in dementia–ERRATUM. J. Int. Neuropsychol. Soc. 16, 737–747. doi: 10.1017/S1355617710000883 19635178

[B56] Tsunga L. P. Hiscox L. V. Halligan S. L. Donald K. A. Fraser A. (2025). Violence exposure and cognitive outcomes among children in low-and middle-income countries (LMICs): a systematic review. Trauma, Violence Abuse 27, 407–428.. doi: 10.1177/15248380251316232 40071810 PMC12953671

[B57] Valencia A. García-Sánchez E. Vernaza-Caucayo M. A. Bejarano-Valencia Y. Sánchez-Vélez J. Jiménez-Polanía A. . (2025). Posttraumatic stress symptoms negatively affect executive functioning: evidence from internally displaced Colombian adolescents. Peace Confl. 32, 120–126. doi: 10.1037/pac0000814

[B58] Veggi S. Roveta F. (2025). Neurodegenerative disorders in criminal offending and cognitive decline among aging inmates. NeuroSci. 6:5. doi: 10.3390/neurosci6010005 39846564 PMC11755462

[B59] Vermeulen R. Schymanski E. L. Barabási A. L. Miller G. W. (2020). The exposome and health: where chemistry meets biology. Science 367, 392–396. doi: 10.1126/science.aay3164 31974245 PMC7227413

[B60] Villanueva-Bonilla C. Londoño-Guzmán D. Ríos-Gallardo Á. Montoya-Arenas D. Gaviria-Gomez A. M. (2024). Effect of a neuropsychological intervention program on executive functions and social cognition in select child witnesses in the Colombian armed conflict. Peace Conf. 30:151. doi: 10.1037/pac0000700

[B61] Volarić N. Šojat D. Volarić M. Včev I. Keškić T. Majnarić L. T. . (2024). The gender and age perspectives of allostatic load. Front. Med. 11:1502940. doi: 10.3389/fmed.2024.1502940 PMC11685202 39741506

[B62] Wadhwa R. Gupta R. Maurya P. K. (2018). Oxidative stress and accelerated aging in neurodegenerative and neuropsychiatric disorder. Curr. Pharm. Des. 24, 4711–4725. doi: 10.2174/1381612825666190115121018 30644343

[B63] Williams D. R. Yu Y. Jackson J. S. Anderson N. B. (1997). Racial differences in physical and mental health: socio-economic status, stress and discrimination. J. Health Psychol. 2, 335–351. doi: 10.1177/135910539700200305 22013026

[B64] Zannas A. S. (2024). Biological aging and mental illness—a vicious cycle? JAMA Psychiatry 81, 433–434. doi: 10.1001/jamapsychiatry.2024.0084 38477905

